# Intravenous Administration of Vitamin C in the Treatment of Herpes Zoster-Associated Pain: Two Case Reports and Literature Review

**DOI:** 10.1155/2020/8857287

**Published:** 2020-12-01

**Authors:** Yao Liu, Mi Wang, Miao-Miao Xiong, Xue-Guang Zhang, Ming Fang

**Affiliations:** ^1^Department of Pain Management, The Affiliated Hospital of Jiangnan University, Wuxi, Jiangsu, China; ^2^Jiangsu Province Key Laboratory of Anesthesiology and Analgesia Application Technology, Xuzhou Medical University, Xuzhou 221004, China; ^3^Department of Anesthesiology, The Affiliated Hospital of Jiangnan University, Wuxi, Jiangsu, China

## Abstract

**Background:**

Herpes zoster (HZ) is an acute inflammatory neurocutaneous disease caused by the reactivation of varicella-zoster virus. It is estimated that the incidence of postherpetic neuralgia following HZ is 10–20%. The leading risk factors of the prognosis are aging and immunity dysfunction. Vitamin C plays a pivoted role in enhancing white blood cell function. Epidemiological evidence and clinical studies have indicated an association between pain and suboptimal vitamin C status. At present, vitamin C has been used as an additional option in the treatment of HZ-associated pain. Despite the current controversy, case reports and randomized controlled studies have indicated that both acute- and postherpetic neuralgia can be dramatically alleviated following intravenous vitamin C infusions. *Case Presentation*. Two patients (male aged 72 and female 78 years) with HZ did not respond well to antiviral therapy and analgesics. Skin lesions in the right groin and front thigh healed after early antiviral therapy, but the outbreak of pain persisted in the male patient. The female patient presented to our clinic with clusters of rashes in the right forehead with severe edema of her right upper eyelid. Because nerve blockade could not be conducted for both patients, intravenous infusion of vitamin C was applied and resulted in an immediate remission of the breakthrough pain in the male patient and cutaneous lesions in the female patient.

**Conclusions:**

The use of vitamin C appears to be an emerging treatment alternative for attenuating HZ and PHN pain. Hence, we recommend the addition of concomitant use of intravenously administered vitamin C into therapeutic strategies in the treatment of HZ-associated pain, especially for therapy-resistant cases. Furthermore, animal studies are required to determine analgesic mechanisms of vitamin C, and more randomized clinical trials are essential to further determine the optimal dose and timing of administration of vitamin C.

## 1. Background

Herpes zoster (HZ) is an acute inflammatory neurocutaneous disease caused by the reactivation of varicella-zoster virus (VZV) that remains latent in the dorsal root ganglia or cranial nerve ganglia after initial infection [1, 2]. Postherpetic neuralgia (PHN), which is the most frequent chronic complication of HZ and the most common peripheral neuropathic pain resulting from infection, is defined as pain persisting more than 30 days after the onset of the rash in the same affected dermatome [3, 4]. Patients with PHN may suffer from continuous or paroxysmal pain, which can be characterized by hyperaesthesia, hyperalgesia, and allodynia [5]. It is estimated that the annual incidence of acute HZ is 2.0–4.6 cases per 1000 persons in Europe [6], and the incidence of PHN following HZ is 10–20%, rising significantly from the age of 50 years [7], while in patients in their 80s, the incidence of PHN is over 30% [8].

Pharmacologic therapy is the first-line treatment for both HZ and PHN. Treatment for HZ is focused on inhibiting viral replication, alleviating pain, and preventing PHN [9–11]. Current medical treatments for patients with PHN include calcium channel blocker, tricyclic antidepressants, and opioid analgesics [12, 13]. Other methods include nerve blockade or modulation, topical therapy, physical therapy, and alternative therapy [14]. Treatment with antivirals within 72 hours of onset of rash has shown a reduction in herpes zoster and its complications [15]. Famciclovir and valacyclovir were preferred to aciclovir for antiviral therapy, and a general preference towards pregabalin is shown for the treatment of increasing severity of pain [16]. Spinal cord stimulation is effective in reducing and preventing PHN but at an increasing cost [17]. Acute zoster pain can be reduced with epidural anesthetics and steroids [18]. Despite several therapeutic modalities for herpes zoster and its complications, the treatment remains a challenge.

It has been confirmed that aging and suppressed cellular immunity are the strongest risk factors for both HZ and PHN [19]. Therefore, enhancing the immune function of patients is an important therapeutic strategy, especially in the elderly frail patients. Vitamin C, also known as L-ascorbic acid, has been widely studied since its discovery and isolation by Szent-Gyorgyi in the 1930s [20, 21]. Vitamin C is an essential micronutrient in many metabolic pathways, acting as a water-soluble antioxidant, and plays a key role in enhancing white blood cell function and promoting protein metabolism and neurotransmitter production [22, 23]. Unlike animals, humans are unable to synthesize this essential vitamin due to a lack of L-gulonolactone oxidase activity, and therefore, it is taken from natural dietary sources or supplements [24].

Epidemiological evidence has indicated an association between several models of pain (musculoskeletal, virus-associated, cancer-related, and postsurgical pain) and suboptimal vitamin C status [25–28]. A community-based case-control study has revealed that lower vitamin C intake significantly increases HZ risk among daily micronutrient intakes [29]. Another study has shown that the concentration of vitamin C in the plasma of PHN patients is lower, and the high sensitivity of PHN patients to vitamin C deficiency may be a permanent factor in the formation of chronic neuropathic pain [26]. Although the current efficacy of vitamin C in the treatment of HZ-associated pain is still controversial, recent reports have shown that vitamin C can exhibit analgesic properties in the treatment of both acute herpetic neuralgia (AHN) and PHN [30, 31]. Herein, we report two patients with AHN who reported an immediate decrease in pain after intravenous administration of vitamin C and review articles about intravenous vitamin C treatment for herpetic neuralgia and PHN. We also analyze the pros and cons of high-dose vitamin C administration and highlight the need for an advanced understanding of the pharmacokinetics of intravenous vitamin C in future studies. Both the patients gave written informed consent for publication of this report.

## 2. Case Report 1

The first case was of a 72-year-old male whose somatic anamnesis was unremarkable and no prior intake of medications. He was hospitalized for localized zoster in the right groin and front thigh for 23 days. Eight days after tooth extraction, the patient developed a local rash with pinching pain and was diagnosed with extensive HZ of dermatomes T12 to L2 by a dermatologist. The oral medication was initially 200 mg celecoxib twice a day, 75 mg pregabalin, and 250 mg of famciclovir three times a day, for 7 days, respectively. The patient-reported visual analogue scale (VAS) score was controlled, no more than 6. On the 19th day after the appearance of the rash, the patient developed severe breakthrough pain, with more than ten attacks per day (about 5–6 outbreaks at night), each episode lasting 5–8 minutes, accompanied by a tremor of the right lower limb. The VAS pain score was 10 in the onset of breakthrough pain and 0 in the resting. The initial dermatologist tried to increase the dosage of pregabalin, but the patient reported intolerable dizzy and lethargy without any alleviation of his breakthrough pain. Therefore, the patient was admitted and prescribed 75 mg pregabalin three times a day, as well as 100 mg tramadol hydrochloride every 12 hours. Two days after hospitalization, the patient reported no reduction of the intensity and number of episodes of breakthrough pain, after which epidural blockade was performed to resolve the pain. However, during the process, breakthrough pain recurred and the patient was unable to keep his lateral position and switched to a supine position; therefore, the treatment was suspended. The patient received repetitive infusions of 4 g of vitamin C in 250 ml of physiological saline solution, without adjusting the dosage of pregabalin and tramadol hydrochloride. On the second night, there were still 5 episodes of breakthrough pain, but the duration of each attack did not exceed 3 minutes, and the VAS dropped to 8. On the fourth day, although the daily attack frequency did not decrease markedly, the duration had dropped to about 1 minute and the VAS was 6. On the seventh day, the second attempt of epidural blockade using 10 ml 1% lidocaine with 40 mg methylprednisolone was performed successfully. Although the patient suffered an attack of breakthrough pain after lying in the lateral position, the pain intensity was tolerable and lasted for merely 1.5 minutes. During the first 24 hours after the epidural blockade, there were 5 times of attacks, each time lasting for no more than 1 minute and the pain intensity remaining, with a VAS of 3-4. On the tenth day after intravenous administration of vitamin C, the patient had only one attack in the daytime, lasting for half a minute, with a VAS of 1-2. The next day, he was totally pain-free and was discharged from the hospital with an oral prescription for 75 mg pregabalin three times a day for two days. At 1-week and 3-month follow-up, there was no pain recurrence.

## 3. Case Report 2

**Figure 1:**
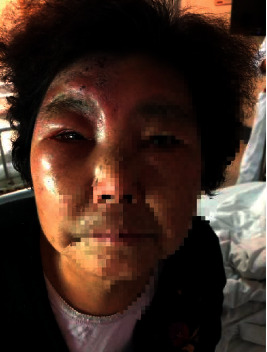
State of the right upper eyelid on the day of hospitalization (13 days after the onset of rash).

**Figure 2:**
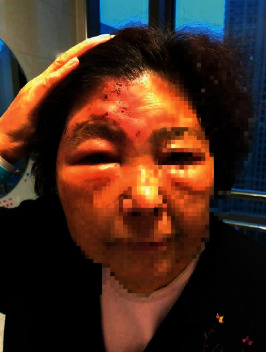
State of the right upper eyelid five days after intravenous administration of vitamin C.

**Figure 3:**
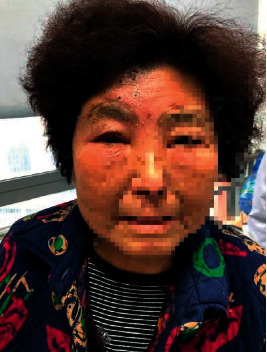
State of the right upper eyelid one week after discharge from hospital (28 days after the onset of rash).

A 78-year-old female patient, with a history of diabetes mellitus and rheumatoid arthritis (treated with metformin, Tripterygium, and total glucosides of paeony accordingly), presented to our clinic with clusters of rashes in the right forehead for 13 days with severe edema on her right upper eyelid (Figure 1). She was diagnosed with acute HZ in the first branch of the right trigeminal nerve. The patient suffered from constant burning pain after the cutaneous eruption, and the intensity on the VAS was 8, with sleep quality seriously affected. She has prescribed 75 mg pregabalin along with paracetamol/tramadol (37.5 mg/325 mg) three times per day. There was no pain relief on the second day. Therefore, paracetamol/tramadol was replaced by 100 mg tramadol hydrochloride every 12 hours. Two days later, the VAS dropped to 6, but her duration of sleep at night still lasted for no more than 3 hours. It was unrealistic to perform supraorbital nerve blockade as her eyelid was severely edematous, and thus, the right stellate ganglion block (SGB) was recommended. However, the patient refused to take SGB. After confirming that there was no contraindication of using vitamin C, a mixture of 250 ml normal saline water and 4 g vitamin C was administered intravenously on the fourth day of hospitalization. However, it did not alleviate the pain, and therefore, vitamin C dose was added up to 8 g the next day. The VAS was reduced to 3 at night, and the patient slept for about 6 hours without pain disruption. After 5 days of treatment, the patient claimed that the pain was unperceivable, and there was a significant improvement in her eyelid edema (Figure 2). After discharge from the hospital, pregabalin and tramadol hydrochloride dose were reduced gradually and stopped within one week, after eyelid edema disappearance (Figure 3). At 3-month follow-up, she continued to be pain-free without any complications.

## 4. Literature Review

### 4.1. Herpes Zoster-Associated Pain and Several Analgesic Mechanisms of Vitamin C

Studies have shown that the decline of cell-mediated immune function plays a critical role in the reactivation of VZV infection and the development of PHN; therefore, investigating the role of immune-relevant micronutrition from the therapeutic point of view is worthwhile [2–4, 32]. VZV remains dormant in the spinal or cranial sensory ganglia after primary infection earlier in life and becomes reactivated afterwards, traveling down the sensory root ganglia to cause damage to peripheral and central neurons, ultimately resulting in an inflammatory immune response [33].

Newly synthesized viral particles can be transported along the axons of all types of sensory neurons, resulting in neuronal necrosis in the affected ganglia and sensory nerves to the skin [1] and loss of the ability to inhibit the transmission of nociceptive pain after peripheral nerve injury, thereby reducing the threshold for nociceptive activation and producing spontaneous ectopic discharge. In addition, VZV-induced neuroinflammation impairs the central pain-suppressing pathway and leads to central sensitization, resulting in an enhanced central response from normal stimulation of peripheral nociceptors, which play an important role in the pathogenesis of PHN [3, 4].

Chen et al. [26] found that plasma vitamin C concentration was lower in PHN patients than in healthy volunteers. They subsequently conducted a randomized, double-blind, placebo-controlled trial and found that short-term intravenous administration of large doses of vitamin C helped attenuate spontaneous pain. Currently, vitamin C has been postulated to alleviate HZ-associated pain through several possible mechanisms of action.

First, recent studies have revealed that VZV-induced peripheral inflammation sensitized nociceptors can produce excessive reactive oxygen species (ROS), which strongly reacts with noxious stimuli, causing the response to peripheral sensitization and thereafter inducing central sensitization in the spinal cord [21, 22, 34]. The high concentration of vitamin C around the immune cells and neurons may explain the result of Chen's study that vitamin C concentration is lower in PHN patients and intravenous administration of vitamin C as a ROS scavenger can exhibit analgesic properties [35]. For HZ, vitamin C can also reduce inflammation by the production of antiviral cytokine and interferon and thus has a direct antiviral effect [36].

Second, enhancing the spinal descending inhibitory pathway is another possible mechanism of vitamin C to reduce HZ-associated pain [37]. One critical mechanism of neuropathic pain in spontaneous pain is disinhibition, which is mediated by the spinal descending inhibitory pathway [38]. Spinal monoamines, including norepinephrine and serotonin, have been known to be involved in the descending inhibition of nociceptive transmission. Noradrenergic fibers from the brainstem terminate in the superficial dorsal horn and release norepinephrine to exert its antinociceptive actions [39, 40]. Vitamin C is a key cofactor of dopamine b-monooxygenase through which dopamine can be converted into norepinephrine. And the conversion is maximally efficient only in cells repleting with external vitamin C [41, 42].

Third, *β*-endorphin has been reported to be able to elevate the threshold of chronic neuropathic pain, and vitamin C can augment the production and release of *β*-endorphin by enhancing the adenylyl cyclase-cyclic adenosine monophosphate system. [43–45]. Besides, one recent study demonstrated elevated levels of tumor necrosis factor (TNF)-a, interleukin-1b (IL-1b), and IL-6 in an animal model of artificially induced neuropathic pain. Furthermore, due to the progression of PHN, elevated levels of IL-8, which is known to be secreted by VZV-infected cells, were verified as a marker and predictor of neuropathic pain [46, 47]. Another recent animal study concerning the influence of vitamin C on the production of TNF-a and IL-6 in ethyl-toxic liver disease showed that, in vitamin C-treated rats, the serum concentration of TNF-a and IL-6 was significantly decreased [48]. Thus, it is proposed that vitamin C can also exhibit analgesic properties by modulating serum levels of the cytokine.

Carr and McCall [49] proposed a novel analgesic mechanism for vitamin C in a literature review. It is well established that vitamin C can act as a cofactor for peptidylglycine *α*-amidating monooxygenase, which is the only enzyme known to amidate the carboxy terminal residue of neuropeptides and peptide hormones [50]. Therefore, vitamin C participates in the amidation of peptides as a cofactor for the biosynthesis of amidated opioid peptides. The proposed mechanism was based on studies that showed a decreased requirement for opioid analgesics in surgical and cancer patients administered with high-dose vitamin C [51, 52].

### 4.2. Application Profile and Curative Effect Analysis of Intravenous Supplement of Vitamin C in the Treatment of HZ-Associated Pain

Nowadays, research on vitamin C in neuropathic pain mainly revolves around its preventive effects on complex regional pain syndrome (CRPS) after fracture [53, 54]. Inflammation after tissue trauma and neuroinflammation of the peripheral nervous system in rats has been shown to induce spontaneous pain [38]. It has been advocated that it may be beneficial to supply and increase plasma concentrations of vitamin C for wrist fracture patients at high risk for CRPS (type I) [55]. Zollinger and colleagues [56] performed a randomized controlled study on the effect of vitamin C on the frequency of reflex sympathetic dystrophy (RSD) in wrist fractures. The results showed that higher vitamin C intake was positively associated with fewer risks of RSD, and the authors assumed that vitamin C could have a similar beneficial effect in other forms of trauma. Since 2000, several scholars in different countries have conducted numerous trials to investigate the role of vitamin C in preventing CRPS after fractures [57]. The American Association of Orthopaedic Surgeons has recommended the use of vitamin C in patients with distal radius fractures to prevent CRPS [54].

Based on the mechanisms previously mentioned in this study, researchers have conducted several studies on the application of vitamin C in HZ-associated pain:

1. Case report: an animal study conducted by Hanck and Weiser [58], published as early as 1985, reported a dose-dependent pain reduction by oral vitamin C in rats. Chen et al. [59] reported in 2006 that a patient with intractable PHN was treated intravenously with 2.5 g/d vitamin C. His spontaneous pain was completely resolved within 1 week, with his plasma vitamin C level increasing to 14.9 mg/L compared to the pretreatment baseline value of 4.9 mg/L. On follow-up examination after 3 months, the patient had no pain recurrence and the plasma vitamin C level was 11.6 mg/L.
2. Subsequently, Schencking et al. [60] in 2009 and Byun and Jeon [61] in 2011 reported 2 cases and 1 case, respectively, of intravenous administration of vitamin C in treating AHN, and both the results showed the positive analgesic effect of vitamin C. The former reported a patient that suffered from the acute phase of HZ with severe pain in the frontooccipital in the forehead area and was treated with intravenous administration of 15 g vitamin C every two days over a period of two weeks. The patient stated a reduction of pain from a VAS score of 8 to total pain-free on the fourth date of infusion. Complete remission of the rash was noted as well. The pain intensity was reduced rapidly and markedly without the application of strong analgesic drugs in the whole course. The latter also reported a patient who suffered from constant aching pain with intermittent, spontaneous sore and shooting pain over the right occipital area. The pain intensity did not decrease after pregabalin and SGB administration, and therefore, intravenous administration of vitamin C was attempted. Immediate pain relief for about 12 hours was noted after the first administration of 4 g vitamin C, and the patient's pain intensity had been maintained at a VAS of 0-1. Five days after discharge, the patient reported a complete resolution of pain and stopped taking the prescription of pregabalin and vitamin C. There was neither relapse of pain at 3-month follow-up nor any complications.
3. Randomized controlled trial: Chen et al. [26] identified a lower plasma vitamin C concentration in patients with PHN in a cross-sectional study of 39 healthy volunteers and 38 PHN patients in 2009. They also found a significant correlation of plasma vitamin C concentrations with the intensity of spontaneous pain but not with brush-evoked pain. Subsequently, they performed a randomized, double-blind, placebo-controlled trial, which revealed that seven days of treatment with vitamin C supplementation effectively increased plasma vitamin C concentrations in PHN patients, and spontaneous pain was decreased by 3.1 on a numerical rating scale (NRS) from baseline. However, this effect was not observed in brush-evoked pain. The authors attributed this difference to different mechanisms that spontaneous pain and brush-evoked pain involved in individual patients.
4. The latest randomized controlled study was conducted by Kim et al. [62] in 2016 to evaluate intravenously administrated vitamin C on AHN and its preventive effects on PHN. They found that compared with the control group (42 cases), there was no significant change in the acute pain score within 4 weeks of hospitalization in the vitamin C treatment group (45 cases). However, there were statistically significant differences after the eighth week, which continued thereafter, and the incidence of PHN was dramatically decreased in the vitamin C treatment group. They concluded that vitamin C supplement exerts no positive influence on acute zoster-associated pain, but it is effective in reducing the incidence of PHN. This brought more controversy to the clinical effect of vitamin C. Similarly, Schencking et al. [30] performed a multicenter, prospective cohort study in Germany to evaluate the safety and efficacy of intravenous vitamin C (7.5 g/d for approximately 2 weeks) in 67 participants with symptomatic HZ. A total of 59 patients (92.2%) improved in their VAS scores, and the mean VAS decreased significantly from baseline values in all visits; dermatologic symptoms of shingles between baseline and follow-up assessments were also statistically significant. The overall incidence of PHN in participants was 6.4% and significantly lower than that reported in previous studies (18%–33%). Nevertheless, the lack of a placebo-control group was the major limitation of this study, and the author proposed that more randomized, placebo-controlled clinical trials should be conducted to confirm these findings.

### 4.3. Dosage and Adverse Reactions of Intravenous Infusion of Vitamin C

Because humans cannot synthesize endogenous vitamin C like most animals, intake from dietary sources or supplements is necessary. Pharmacokinetic studies have shown that vitamin C concentrations are tightly regulated through renal resorption and higher oral intake (>100 mg/d) for an adult can barely result in higher absorptivity [63, 64]. Therefore, short-term therapeutic plasma concentration for HZ-associated pain can only be achieved by parenteral administration [65]. Currently, the administration of vitamin C via the intravenous route is widely used in clinical studies, with the daily dosage for treatment varying significantly in different studies. The optimal dose has to be determined through stronger evidence in the future.

There is no consensus on the metabolic process and transformation mode of intravenously administered vitamin C in vivo although it may be the least toxic of all vitamins. The adverse effects of the use of high-dose intravenous vitamin C reported from available data are mostly minor. Sebastian et al. [66] surveyed attendees at annual Complementary and Alternative Medicine (CAM) Conferences in 2006 and 2008, queried for side effects, compiled published cases, and analyzed FDA's Adverse Events Database. A total of 11233 patients in 2006 and 8876 patients in 2008 (20109 total) accepted intravenous vitamin C therapy. The average dose was 28 g every 4 days. Available data revealed that, out of 9328 patients, 101 had minor side effects, including lethargy, fatigue, vein irritation, and mental status change, and 2 patients with glucose-6-phosphate dehydrogenase (G6PD) deficiency died of intravascular hemolysis.

Besides, the metabolic end-product of vitamin C metabolism is oxalate. Patients with renal impairment have been reported to develop oxalate nephropathy when given gram doses of intravenous vitamin C [67]. As for patients with renal failure, long-term high-dose intravenous use increases plasma oxalate concentrations and results in increased urinary oxalate in patients receiving total parenteral nutrition. A high dose of vitamin C has also been proved to elevate the excretion of calcium, iron, and manganese in the urine, potentially increasing the risk of urinary stone formation [68]. Nevertheless, most studies indicated that the administration of vitamin C in patients with normal renal function is unlikely to cause any severe damage. High-dose oral or intravenous vitamin C should be used cautiously in patients with preexisting renal insufficiency. Physicians should also be alert to potential interactions of high-dose vitamin C with conventional medicine and alternative medicine. Importantly, physicians should be cognizant of potential adverse or other unexpected effects. We recommend starting with a low dose and slow intravenous infusion and detection of renal function and G6PD levels before treatment.

## 5. Conclusion

In summary, low plasma levels of vitamin C detected in PHN patients may be due to the excessive oxygen-free radicals caused by a varicella-zoster infection in the early stage. During this process, vitamin C utilization is increased as it functions physiologically as a water-soluble antioxidant by its high reducing power [69]. Vitamin C deficiency has been noted in patients with various painful diseases, such as orthopedic pain, virus-associated pain, and cancer-related pain [26, 70, 71]. Moreover, vitamin C plays a key role in the function of leukocytes, protein metabolism, and the production of neurotransmitters [72, 73]. High vitamin C concentration has been found around immune and nerve cells, indicating the possible positive role of vitamin C in HZ-associated pain. Based on the literature, patients with viral infections exhibit vitamin C deficiencies, which play a critical role in the pathogenesis of herpes infections and the development of PHN. Intravenous vitamin C therapy has not been widely used in patients with AHN or PHN because its beneficial effects on disease conditions are unproven. This review recommends vitamin C treatment as an option when patients do not respond well to conventional therapies.

So far, most studies have been performed in patients with orthopedic trauma and cancer pain [74, 75]. Possible mechanisms of action of vitamin C have been elucidated, making its therapeutic effects biologically plausible for the first time. We expect more rigorous studies to confirm that high-dose intravenous vitamin C may become a safe and effective adjunctive therapy for acute and chronic pain relief in diverse groups of patients, especially in the early stage of varicella-zoster virus infection. Future researches are necessary to ascertain the optimal dosage, interval, and periods of vitamin C administration to achieve the desired therapeutic or preventive effect on HZ-associated pain.

## Data Availability

Data can be made available upon request to the corresponding author.
